# Severity, Progress, and Related Factors of Mood Disorders in Patients with Coronary Artery Disease: A Retrospective Study

**DOI:** 10.3390/healthcare8040568

**Published:** 2020-12-16

**Authors:** Changbae Lee, Sang Cheol Lee, Yeon Seob Shin, Sangwoo Park, Ki Bum Won, Soe Hee Ann, Eun Jae Ko

**Affiliations:** 1 Department of Physical Medicine and Rehabilitation, Ulsan University Hospital, College of Medicine, University of Ulsan, Ulsan 44033, Korea; pclucky7@gmail.com (C.L.); lsc890@naver.com (S.C.L.); mindovermood@naver.com (Y.S.S.); 2 Department of Cardiology, Ulsan University Hospital, College of Medicine, University of Ulsan, Ulsan 44033, Korea; warya7@naver.com (S.P.); kbwon99@naver.com (K.B.W.); ash@uuh.ulsan.kr (S.H.A.); 3 Department of Rehabilitation Medicine, Asan Medical Center, College of Medicine, University of Ulsan, Seoul 05505, Korea

**Keywords:** depression, anxiety, coronary artery disease, cardiopulmonary exercise test

## Abstract

Patients with coronary artery disease (CAD) are more likely to experience depression and anxiety, which, in turn, are risk factors for CAD. The Beck depression inventory (BDI) and Beck anxiety inventory (BAI) were applied for mood evaluation during hospitalization and again 3 months after discharge in 118 patients with CAD, and cardiopulmonary exercise tests were conducted in the outpatient department. Of the patients diagnosed with CAD, 40 (33.9%) had depressive moods, and 51 (43.2%) had anxious moods. A family history of CAD, low Korean activity scale index (KASI), and use of beta-blockers were independent factors causing depressive mood, while lower left ventricular ejection fraction (LVEF) and low KASI score were independent factors causing anxious mood. A considerable number of patients (35.7% with depressive mood and 25.0% with anxious mood) still had emotional problems after 3 months of discharge. The change values of BDI were associated with lower LVEF and longer hospital stay, while those of BAI was associated with a longer hospital stay. Since some patients had depressive and anxious moods after three months of discharge, evaluating and treating them are essential.

## 1. Introduction

Coronary artery disease (CAD) is the global leading cause of death [1]. In addition to traditional risk factors, such as male sex, smoking, and hyperlipidemia, which lead to a reduction in the coronary artery flow, depression and anxiety are also risk factors associated with CAD [2,3,4,5,6,7]. When there is a negative mood change, biological mechanisms cause alterations in autonomic nervous system activity, catecholamine levels, and inflammatory activity, and cause endothelial and platelet dysfunction [4].

Patients with acute myocardial infarction (MI) are more likely to experience depression than the general population; particularly in the United States, this proportion can be about three times higher [8,9]. Furthermore, the anxiety prevalence rate in patients with CAD is almost twice (36%) that of the general population in the United States [10]. Depression and anxiety in patients with CAD are associated with increased mortality, morbidity, and recurrence rate [11,12,13,14,15,16,17]. Therefore, the American Heart Association (AHA) recommends routine screening for depression in patients with CAD and has proposed that effective treatment for depression could improve health outcomes [18].

Several studies have discussed factors related to mood disorders in patients with CAD. Walters et al. found that living alone, experiencing chest pain, disability due to pain and discomfort, challenges in performing usual activities, younger age, problems in close relationships, diabetes mellitus (DM), and female sex were causational factors associated with a higher risk of depression in patients with CAD [19]. In addition, experiencing chest pain increases the risks of both depression and anxiety in patients with CAD [20]. However, since most studies focused on the factors associated with mood disorders, the progress and severity of mood disorders in patients with CAD are not well evaluated. This study aims to evaluate the severity, progress, and factors related to mood disorders in patients with CAD.

## 2. Materials and Methods

### 2.1. Study Design and Patients

This retrospective study enrolled patients referred for cardiac rehabilitation after a percutaneous coronary intervention (PCI) for established CAD in Ulsan University Hospital between 1 January 2019 and 31 December 2019. The subjects met the following inclusion criteria: (a) diagnosed with acute MI, including ST-elevation MI (STEMI), non-ST-elevation MI (NSTEMI), and unstable angina; (b) age above 18 years; and (c) evaluated for depression and anxiety during admission. The exclusion criteria were as follows: (a) difficulty in undergoing the cardiopulmonary exercise tests (CPET) due to gait disturbance related to physical abnormalities such as stroke or a general deconditioned state; and (b) suffering from psychiatric diseases that would interfere with the performance in the CPET. The research protocol was approved by the Ethical Committee of Ulsan University Hospital (ref number: 2020-01-018).

### 2.2. Measurement of Depression and Anxiety

The Beck depression inventory (BDI) is a self-reported questionnaire comprising 21 items that evaluate the symptoms and characteristic attitudes of depression [21]. This assessment tool was intended to assess the level of depressive symptoms, including lack of motivation, loss of interest, depressive thoughts, and dysphoric mood. The BDI was scored using a 4-point Likert scale from 0 to 3 for each item; the total BDI score was 63. The BDI results were classified as follows: healthy, up to 13 points; a minimal level of depression, 14–19 points; mild depression, 20–28 points; and severe depression, 29–63 points. In this study, patients scoring 13 or less were classified into the normal group, and those scoring 14 or more were classified into the depression group.

The Beck anxiety inventory (BAI) consists of a questionnaire comprising 21 items and is used to evaluate the presence and severity of symptoms related to anxiety within a week [22]. It was developed to differentiate between behavioral, physiological, and emotional symptoms and is a simple evaluation tool focused on the somatic symptoms of anxiety. Additionally, this inventory can be used to assess the progress of anxiety treatment [23]. The BAI was used to evaluate symptoms such as nervousness, the inability to relax, and dizziness, and each item was scored on a 4-point Likert scale from 0 to 3 [24]; the total BAI score was 63. BAI scores were classified as minimal anxiety, 0–7 points; mild anxiety, 8–15 points; moderate anxiety, 16–25 points; and severe anxiety, 26–63 points. In this study, patients scoring less than 7 points were classified into the normal group, and those scoring above 7 points were classified into the anxiety group.

The BDI and BAI evaluations were conducted during hospitalization and were repeated 3 months after discharge. The differences between the test scores of the two periods were calculated and considered as the change values of depressive and anxious moods.

### 2.3. CPET

The CPET is the most widely used clinical evaluation method for the function and capacity of coronary arteries and involves non-invasively applying physiological stress to the cardiac system [25]. The CPET was conducted at the second follow-up in the outpatient department. It was performed with the Quark CPET (COSMED, Rome, Italy) system and OMNIA software, using the Bruce or modified Bruce protocol on the treadmill. The examination was supervised by an experienced physical therapist, a nurse, and a rehabilitation doctor. During the examination, the breathing gas was analyzed through a sealed facemask. Gas, pressure, and volume were automatically calibrated before the test. In addition, the rating of perceived exertion was expressed on the Borg’s scale (from 6 to 20). The CPET was conducted with symptom limitation, so the subjects could terminate the test when they found it difficult to proceed. Furthermore, the respiratory quotient was evaluated to assess whether the patients had undergone a sufficient amount of intense exercise. The results of the test included peak volume of oxygen consumed by the body per minute per weight (VO_2peak_/kg), maximal metabolic equivalent task (MET), peak heart rate (peak HR), peak systolic blood pressure (peak SBP), peak diastolic blood pressure, rate pressure product (RPP) values, anaerobic threshold (AT), and ventilatory equivalent for carbon dioxide.

### 2.4. Other Measurements

Demographics and baseline data were evaluated, including sex, age, the clinical presentation of CAD (STEMI, NSTEMI, or unstable angina), the number of diseased vessels, the number of treated vessels, left ventricular ejection fraction (LVEF), previous history of CAD, previous history of depression and anxiety, family history of CAD, and underlying diseases such as hypertension, DM, and dyslipidemia. Smoking status (never, smoker, or ex-smoker), alcohol consumption, education level, and length of hospital stay were reviewed from the patients’ medical records. The working status was categorized as working or not working. The residence type was classified into two groups: married or living with a partner; and single, separated, or widowed. The body mass index (BMI) score was classified as follows: normal, <23; overweight, 23–24.9; obesity, 25–29.9; and extreme obesity, ≥30 [26]. Physical activity was classified into three categories: high physical activity for moderate-intensity exercise of ≥300 min/week, medium physical activity of ≥150 min for <300 min per week, and low physical activity for <150 min per week [27]. The functional status was assessed during admission using the Korean activity scale index (KASI; 0–79), which is a simple technique to evaluate patients’ quality of life by including aspects such as daily life, sports-related activities, housework, and sex life [28]. Additionally, the use of beta-blockers at discharge was examined through chart review.

### 2.5. Statistical Analysis

To evaluate the related factors of depressive or anxious moods, Student’s *t*-test or Mann–Whitney U test was used for continuous variables, and Pearson’s chi-squared test or Fisher’s exact test was used for categorical variables. The related factors of depressive and anxious moods were analyzed with univariate analysis, and the covariates that were statistically significant on the univariate analysis were analyzed with the multiple logistic regression with forward conditioning to identify the causative factors of depressive or anxious moods. Results of the initial and second assessments of depressive and anxious moods were evaluated using Fisher’s exact test. The relationship between the change values of emotional distress and the baseline characteristics was evaluated using Pearson’s correlation analysis. Statistical analysis was performed using SPSS for Windows (version 24.0; SPSS Inc., Chicago, IL, USA). All reported *p*-values were two-sided, and *p*-values < 0.05 were considered significant for all tests.

## 3. Results

### 3.1. Baseline Characteristics of the Patients

A total of 118 subjects were included, with a mean age of 58.5 ± 11.0 years. Of the total subjects, 33 (28%), 32 (27.1%), and 53 (44.9%) patients were diagnosed with ST-elevation MI, non-ST-elevation MI, and unstable angina, respectively. None of the patients had been previously diagnosed with depression or anxiety, whereas 13 patients (11.0%) had family histories of CAD. On applying the BDI, 40 patients (33.9%) had depressive moods. Further, when the BAI was applied, 51 patients (43.2%) had anxious moods. The severity of depressive and anxious moods and the baseline characteristics regarding the severity of the CAD, smoking status, alcohol consumption, physical activity, residence type, education level, working status, medication, and length of hospital stay are presented in Table 1.

### 3.2. Related Factors Associated with Depressive and Anxious Moods in Patients with CAD

Table 2 shows the associations between the baseline characteristics and the depressive and anxious moods. The numbers of patients with (BDI ≥ 14) and without (BDI ≤ 13) depressive moods were 40 (33.9%) and 78 (66.1%), respectively. The numbers of patients with (BAI ≥ 8) and without (BAI ≤ 7) anxious moods were 51 (43.2%) and 67 (56.8%), respectively. Depressive moods were related to the diagnosis of the patient, lower LVEF, presence of a family history of CAD, smoking status, low levels of physical activity, low KASI score, use of beta-blockers, and longer hospital stays. Anxious moods were related to the diagnosis of the patient, lower LVEF, low KASI score, use of beta-blockers, and longer hospital stays.

Table 3 shows the association between the results of CPET and depressive and anxious moods. Forty-one (34.7%) patients underwent CPET. The average period from discharge to follow-up in the outpatient department was 97.9 ± 45.1 days. Patients with depression showed significantly lower peak SBPs than those without depression. Although not statistically significant, VO_2peak_/kg, METs, peak HR, RPP, and AT tended to be lower in patients with depression and anxiety than in those without mood disorders.

### 3.3. Causative Factors of Depressive and Anxious Moods in Patients with CAD

The causative factors of depressive and anxious moods are shown in Table 4 and Table 5. The presence of a family history of CAD, lower KASI score and use of beta-blocker were found to be independent risk factors for depressive mood. Meanwhile, lower LVEF and KASI values were the independent risk factors for anxious moods.

### 3.4. Follow-Up Data of Depressive and Anxious Moods

**Figure 1:**
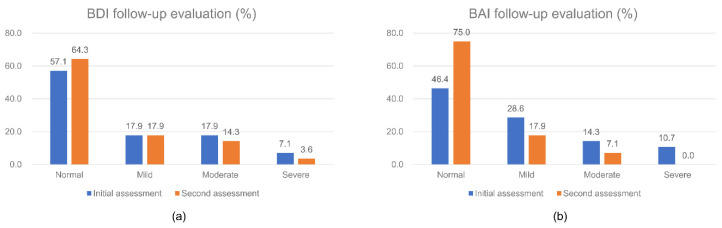
Follow-up evaluations of mood using (**a**) Beck depression inventory (BDI), and (**b**) Beck anxiety inventory (BAI).

After discharge, 28 (23.7%) patients underwent follow-up evaluation for BDI and BAI after 3 months. The changes in BDI and BAI at 3 months compared to the baseline were not statistically significant (*p* = 0.942 and 0.108, respectively) (Figure 1). Three months after discharge, 10 patients (35.7%) still had depressive moods, and 7 patients (25.0%) still had anxious moods.

When the relationship between the change values of emotional distress and the baseline characteristics was evaluated in these 28 patients, lower LVEFs and longer hospital stays were correlated with larger change values of depressive moods. Furthermore, longer hospital stays were correlated with larger change values of anxious moods (Table 6).

## 4. Discussion

This study evaluated the severity, progress, and factors related to mood disorders in patients with CAD. Forty (33.9%) patients had depressive moods, and 51 (43.2%) patients had anxious moods. Depressive moods were related to the diagnosis of the patient, lower LVEF, presence of a family history of CAD, smoking status, low levels of physical activity, low KASI score, use of beta-blockers, and longer hospital stays. Anxious moods were related to the diagnosis of the patient, low LVEF, low levels of physical activity, low KASI score, use of beta-blockers, and longer hospital stays. Furthermore, patients with depression showed significantly lower peak SBPs than those without depression. When the causational factors of depressive and anxious moods in patients with CAD were analyzed, the presence of a family history of CAD, lower KASI score, and use of beta-blocker were independent factors for depression, and lower LVEF and KASI were independent factors for anxiety in patients with CAD when analyzed with regression analysis. Three months after discharge, 10 (35.7%) and 7 (25.0%) patients had depressive and anxious moods, respectively. The change values of BDI were associated with lower LVEF and longer hospital stay, and those of BAI were associated with a longer hospital stay. When the related factors of the mood disorders were investigated, the diagnoses of STEMI and NSTEMI, rather than unstable angina, were associated with depressive and anxious moods. This may be attributable to the higher severity of the clinical symptoms of STEMI and NSTEMI compared to those of unstable angina. Furthermore, longer hospital stays were associated with depressive and anxious moods, probably because longer stays were a greater cause of worry. In a previous study, a correlation between the postoperative length of stay and anxiety after coronary artery bypass graft surgery was reported [29]. Moreover, a family history of CAD also led to a depressive mood, and the diagnosis of the disease tends to be more stressful in patients with a family history of CAD than in those without.

Patients with unhealthy living habits (smoking, lower level of physical activity, and lower KASI raw score) had a greater tendency to suffer from depressive moods. Indeed, previous studies have confirmed that smoking is an independent predictor for depression in CAD patients [30]. In addition, a systematic review confirmed that patients with CAD and depressive mood had a significantly lower probability of smoking cessation [31]. Therefore, education and management of smoking cessation is important, and if patients are unable to quit smoking, it is likely to become a vicious cycle. Furthermore, lower LVEF was related to both depression and anxiety, which corresponds to the results of a previous study that showed a correlation between LVEF and depression [32]. Moreover, a lower EF is expected to be associated with depression because it may accompany a decline in the physical condition, decrease in the quality of life, and difficulties in social function or occupational activities [33]. In addition, tumor necrosis factor-alpha (TNFα), a cytokine that is increased in heart failure, can biologically increase the incidence of depression [34].

The use of beta-blockers at the time of discharge was associated with depression and anxiety. The side effects of the drug or difference in disease severity may be considered as the cause of depression. However, this finding was different from that of previous studies [35,36] that have shown no statistical correlation between the use of beta-blockers and depression at least six months after the initiation of drug use.

Interestingly, sex, age, alcohol, residence type, education level, and working status did not show associations with depressive and anxious moods. In contrast, Shankman et al. [37] suggested that alcohol was associated with depression in patients with CAD. Moreover, Mallik et al. [38] reported higher rates of depression in younger (≤60 years old) patients and women, and Spijkerman et al. [39] reported that patients with previous depression and CAD history, socially isolated patients, and women have a higher incidence of depression. The contradictory results in this study with respect to those in previous studies may be attributable to the differences in the measurement tools used (patient health questionnaire [37], and Primary Care Evaluation of Mental Disorders Brief patient health questionnaire [38]) and different disease categories (broad diseases which need cardiac catheterization [37], acute MI only [38,39]), compared to our study, which used BDI as a depression measurement tool in patients with acute MI and unstable angina. However, in the AHA, it is reported that there are differences in causes and symptoms according to sex [40], and adverse outcomes are more likely in women than men [41]. Therefore, evaluating and managing mood disorders is important in women, even though sex and mood problems did not show associations in this study.

On analyzing the differences in the CPET, patients with depression showed lower peak SBPs than those without depression. Furthermore, VO_2peak_/kg, METs, peak HR, RPP, and AT tended to be lower in patients with depression and anxiety than in patients without mood disorders. These results were similar to those of a previous study that showed that poor performance in CPET corresponded to a higher incidence of depression [39]. Behavioral changes due to mood changes may affect medication compliance, diet, exercise, and smoking, which may result in worse CPET results in patients with depressive and anxious moods than in patients without them.

In addition to the relation or association, multivariate logistic regression was performed to confirm the causation of the mood disorder. We found that a family history of CAD, lower KASI score, and use of beta-blockers were causative factors of depressive mood when analyzed with regression analysis. Further, the lower the values of KASI and LVEF, the more anxious the mood of the patients. It implies that healthy living habits that maintain a high level of physical activity could reduce the risk of depression after CAD. Similarly, maintaining a high level of physical activity and good heart function, which could be achieved from regular aerobic exercise, could reduce the risk of anxiety after CAD.

When patients with CAD were discharged, a considerable number of patients (35.7% with depressive mood and 25.0% with anxious mood) still had emotional problems 3 months later; this may influence atherosclerotic progression [42,43] and increase the risk of CAD [44,45]. In a study by van Melle et al., depression was associated with a 2.7-fold increase in the risk of cardiac-related death, a 2.3-fold increase in all-cause death, and a 1.6-fold increase in cardiovascular events [46]. Moreover, in the statement by the AHA, depression was identified as a risk factor for poor prognosis in patients with acute coronary syndrome [2]. For these reasons, psychological problems that may occur after CAD require constant and close follow-up evaluations and treatment. Pharmacological therapy has shown significant effects in patients with CAD and depression, and anxiety [47]. For example, bupropion not only reduces depressive moods but also helps in smoking cessation and reducing nicotine withdrawal, which ultimately reduces the smoking problem [48,49]. Furthermore, psychosocial interventions can be used to obtain modest reductions in depression after CAD [50]. Psychotherapy can be used as an alternative to antidepressants [51], and cognitive behavioral therapy can also help to improve depression [52].

Three months after discharge, some patients showed greater improvements in emotional distress, while others showed fewer improvements. Longer hospital stays were found to be correlated with larger change values of depressive and anxious moods. Additionally, lower EFs showed correlations with larger change values of depressive moods in our results. Lower EFs may be associated with increments of TNFα, which causes depressive moods; the level of TNFα may decrease after the treatment of heart disease, leading to an improvement in the mood [34].

This study has several limitations that should be mentioned. First, this study has fundamental limitations as it is a retrospective study based on information that was previously investigated. Second, the number of patients who underwent CPET was small. Third, there could be a selection bias since patients who did not undergo mood evaluation were not included. Fourth, only one follow-up evaluation was conducted, and the number of patients with follow-up evaluation results was small. Fifth, none of the patients had a previous history of depression and anxiety; therefore, the impact of this factor on the mood disorder after CAD could not be discussed. Lastly, the effects of cardiac rehabilitation programs on mood disorders were not evaluated in this study.

## 5. Conclusions

This study showed that depressive and anxious moods were associated with many factors; the presence of a family history of CAD, lower KASI score, and use of beta-blocker were independent factors causing depressive mood, and lower LVEFs and lower KASI score were independent factors causing anxious mood. Treatment of these mood disorders is essential since a considerable number of patients have depressive and anxious moods three months after discharge that could negatively affect their prognosis. Furthermore, considering the increasing global burden of depression, both health care centers and the government should pay attention to this issue.

## Figures and Tables

**Table 1:** Baseline characteristics of the patients.

Characteristics	Value
Sex (male; female)	95 (80.5); 23 (19.5)
Age	58.5 ± 11.0
Diagnosis (STEMI; NSTEMI; unstable angina)	33 (28.0); 32 (27.1); 53 (44.9)
Number of diseased vessels	1.8 ± 0.9
Number of treated vessels	1.1 ± 0.6
LVEF (%)	54.8 ± 11.6
Hypertension	61 (51.7)
Diabetes mellitus	43 (36.4)
Dyslipidemia	38 (32.2)
Previous history of CAD	30 (25.4)
Previous history of depression or anxiety	0 (0.0)
Family history of CAD	13 (11.0)
Smoking status (never; smoker; ex-smoker)	49 (41.5); 44 (37.3); 25 (21.2)
Alcohol	62 (52.5)
Physical activity (high; medium; low)	21 (17.8); 41 (34.7); 56 (47.5)
KASI raw score	43.7 ± 13.7
BMI (normal; overweight; obese; extremely obese)	32 (27.1); 25 (21.2); 54 (45.8); 7 (5.9)
Residence type (married; single)	100 (84.7); 18 (15.3)
Education level of raw years	12.0 ± 3.6
Working status (working; not working)	77 (65.3); 41 (34.7)
Use of beta-blockers	60 (50.8)
Length of hospital stay	3.6 ± 2.6
Depressive mood (normal; mild; moderate; severe)	78 (66.1); 18 (15.3); 16 (13.6); 6 (5.1)
Anxious mood (normal; mild; moderate; severe)	67 (56.8); 31 (26.3); 12 (10.2); 8 (6.8)

Values are presented as mean ± SD or number (%). STEMI: ST-elevation myocardial infarction, NSTEMI: non-ST-elevation myocardial infarction, LVEF: left ventricular ejection fraction, CAD: coronary artery disease, KASI: Korean activity scale index, BMI: body mass index, BDI: Beck depression inventory, BAI: Beck anxiety inventory.

**Table 2:** Factors related to depressive and anxious moods in patients with CAD.

		Depression (+) (*n* = 40)	Depression (−) (*n* = 78)	*p*-Value	Anxiety (+) (*n* = 51)	Anxiety (−) (*n* = 67)	*p*-Value
Sex	Male	32 (80.0)	63 (80.8)	0.92	37 (72.5)	58 (86.6)	0.06
	Female	8 (20.0)	15 (19.2)		14 (27.5)	9 (13.4)	
Age		58.0 ± 13.0	58.8 ± 9.8	0.72	58.2 ± 11.7	58.8 ± 10.4	0.75
Diagnosis	STEMI	15 (37.5)	18 (23.1)	<0.001 *	20 (39.2)	13 (19.4)	0.02 *
	NSTEMI	14 (35.0)	18 (23.1)		15 (29.4)	17 (25.4)	
	Unstable angina	11 (27.5)	42 (53.8)		16 (31.4)	37 (55.2)	
Number of vessels involved		1.8 ± 0.8	1.7 ± 0.8	0.48	1.9 ± 0.8	1.7 ± 0.8	0.19
Number of stent-insertion vessels		1.0 ± 0.4	1.1 ± 0.6	0.52	1.1 ± 0.6	1.1 ± 0.6	0.85
LVEF (%)		50.6 ± 13.0	57.0 ± 10.1	0.004 *	50.3 ± 13.2	58.3 ± 8.7	0.001 *
Hypertension		21 (52.5)	40 (51.3)	0.90	23 (45.1)	38 (56.7)	0.21
Diabetes mellitus		17 (42.5)	26 (33.3)	0.33	19 (37.3)	24 (35.8)	0.78
Dyslipidemia		17 (42.5)	21 (26.9)	0.09	20 (39.2)	18 (26.9)	0.16
Previous history of CAD		13 (32.5)	17 (21.8)	0.20	15 (29.4)	15 (22.4)	0.39
Previous history of depression or anxiety		0 (0.0)	0 (0.0)	-	0 (0.0)	0 (0.0)	-
Family history of CAD		8 (20.0)	5 (6.4)	0.03 *	6 (11.8)	7 (10.4)	0.82
Smoking status	Never	12 (30.0)	37 (47.4)	<0.001 *	22 (43.1)	27 (40.3)	0.43
	Smoker	20 (50.0)	24 (30.8)		21 (41.2)	23 (34.3)	
	Ex-smoker	8 (20.0)	17 (21.8)		8 (15.7)	17 (25.4)	
Alcohol		22 (55.0)	40 (51.3)	0.70	25 (49.0)	37 (55.2)	0.50
Physical activity	High	5 (12.5)	16 (20.5)	0.003 *	10 (19.6)	11 (16.4)	0.57
	Medium	12 (30.0)	29 (37.2)		15 (29.4)	26 (38.8)	
	Low	23 (57.5)	33 (42.3)		26 (51.0)	30 (44.8)	
KASI raw score		39.6 ± 13.5	45.8 ± 13.4	0.02 *	39.9 ± 13.7	46.6 ± 13.1	0.01 *
BMI	Normal	7 (17.5)	25 (32.1)	0.24	12 (23.5)	20 (29.9)	0.59
	Overweight	10 (25.0)	15 (19.2)		13 (25.5)	12 (17.9)	
	Obesity	19 (47.5)	35 (44.9)		22 (43.1)	32 (47.8)	
	Extreme obesity	4 (10.0)	3 (3.8)		4 (7.8)	3 (4.5)	
Residence type	Married or living with partner	31 (77.5)	69 (88.5)	0.12	42 (82.4)	58 (86.6)	0.53
	Single, separated, or widowed	9 (22.5)	9 (11.5)		9 (17.6)	9 (13.4)	
Education level raw year		11.3 ± 3.6	12.3 ± 3.6	0.07	11.8 ± 4.0	12.0 ± 3.2	0.90
Working status	Working	22 (55.0)	55 (70.5)	0.09	30 (58.8)	47 (70.1)	0.20
	Not working	18 (45.0)	23 (29.5)		21 (41.2)	20 (29.9)	
Use of beta-blockers		28 (70.0)	32 (41.0)	0.003 *	32 (62.7)	28 (41.8)	0.02 *
Length of hospital stay		4.3 ± 3.5	3.2 ± 2.0	0.02 *	4.1 ± 2.5	3.2 ± 2.7	0.002 *

Values are presented as mean ± SD or number (%). STEMI: ST-elevation myocardial infarction, NSTEMI: non-ST-elevation myocardial infarction, LVEF: left ventricular ejection fraction, CAD: coronary artery disease, KASI: Korean activity scale index, BMI: body mass index. * *p* < 0.05.

**Table 3:** Associations between the results of symptom-limited exercise tolerance test and depressive and anxious symptoms.

	Depression (+) (*n* = 16)	Depression (−) (*n* = 25)	*p*-Value	Anxiety (+) (*n* = 20)	Anxiety (−) (*n* = 21)	*p*-Value
VO_2peak_/kg (mL/kg/min)	25.7 ± 5.0	27.2 ± 5.2	0.630	26.3 ± 5.4	27.0 ± 5.0	0.664
METs	7.3 ± 1.4	7.8 ± 1.5	0.536	7.5 ± 1.5	7.8 ± 1.4	0.551
RPE on Borg’s scale	15.5 ± 1.1	15.2 ± 1.1	0.682	15.3 ± 1.1	15.3 ± 1.2	0.816
Peak HR (bpm)	145.3 ± 19.4	153.5 ± 26.2	0.162	143.6 ± 23.3	158.0 ± 23.7	0.057
Peak SBP (mmHg)	172.5 ± 20.9	190.2 ± 28.0	0.022 *	176.5 ± 26.0	192.4 ± 26.2	0.058
Peak DBP (mmHg)	83.4 ± 10.0	84.1 ± 8.8	0.807	83.2 ± 9.3	84.6 ± 9.0	0.622
RPP (mmHg·bpm)	22,837.3 ± 5558.6	25,789.1 ± 6127.3	0.074	23,241.0 ± 6735.6	26,388.6 ± 4991.7	0.054
AT (mL/min)	1402.5 ± 233.8	1497.3 ± 362.8	0.567	1408.7 ± 301.3	1523.0 ± 348.7	0.269
VE/VCO_2_	30.1 ± 7.4	26.3 ± 4.1	0.101	28.3 ± 5.7	26.7 ± 5.4	0.354
RQ	1.18 ± 0.09	1.21 ± 0.10	0.248	1.19 ± 0.07	1.21 ± 0.11	0.375

Values are presented as mean ± SD. VO_2peak_/kg: peak volume of oxygen consumed by the body per minute per kg body weight, MET: maximal metabolic equivalent task, RPE: rating of perceived exertion, HR: heart rate; SBP: systolic blood pressure, DBP: diastolic blood pressure, RPP: rate pressure product; AT: anaerobic threshold, VE/VCO_2_: ventilatory equivalent for carbon dioxide, RQ: respiratory quotient. * *p* < 0.05.

**Table 4:** Causational factors for depressive mood in patients with CAD.

	Univariate Analyses			Multivariate Analyses		
Factor	Odds Ratio	95% CI	*p*-Value	Odds Ratio	95% CI	*p*-Value
Diagnosis						
STEMI	-	-	0.028 *			
NSTEMI	0.933	0.351–2.483	0.890			
UA	0.314	0.121–0.816	0.017 *			
LVEF (%)	0.953	0.921–0.986	0.006 *			
Family history of CAD	3.650	1.108–12.023	0.033 *	4.050	1.158–14.166	0.029 *
Smoking status						
Smoker	-	-	0.106			
Ex-smoker	0.389	0.161–0.939	0.036 *			
Non-smoker	0.565	0.202–1.580	0.276			
Physical activity						
Low	-	-	0.273			
Medium	0.594	0.252–1.400	0.234			
High	0.448	0.144–1.397	0.167			
KASI raw score	0.967	0.939–0.996	0.024 *	0.967	0.937–0.997	0.034 *
Use of beta-blockers	3.354	1.488–7.562	0.004 *	3.022	1.295–7.052	0.011 *
Length of hospital stay	1.172	0.995–1.380	0.058			

STEMI: ST-elevation myocardial infarction, NSTEMI: non-ST-elevation myocardial infarction, UA: unstable angina, LVEF: left ventricular ejection fraction, CAD: coronary artery disease, KASI: Korean activity scale index. * *p* < 0.05.

**Table 5:** Causational factors of anxious mood in patients with CAD.

	Univariate Analyses			Multivariate Analyses		
Factor	Odds Ratio	95% CI	*p*-Value	Odds Ratio	95% CI	*p*-Value
Diagnosis						
STEMI	-	-	0.022 *			
NSTEMI	0.574	0.214–1.535	0.268			
UA	0.281	0.113–0.700	0.006 *			
LVEF (%)	0.937	0.903–0.972	0.000 *	0.940	0.906–0.976	0.001 *
KASI raw score	0.963	0.936–0.991	0.010 *	0.969	0.940–0.998	0.036 *
Use of beta-blockers	2.346	1.112–1.951	0.025 *			
Length of hospital stay	1.149	0.974–1.354	0.099			

STEMI: ST-elevation myocardial infarction, NSTEMI: non-ST-elevation myocardial infarction, UA: unstable angina, LVEF: left ventricular ejection fraction, KASI: Korean activity scale index. * *p* < 0.05.

**Table 6:** Relationship between the change values of emotional distress and the baseline characteristics.

		r (*p*-Value)
Change values of BDI	LVEF (%)	−0.402 (0.034)
	Length of hospital stay	0.576 (0.001)
Change values of BAI	Length of hospital stay	0.481 (0.010)

BDI: Beck depression inventory, BAI: Beck anxiety inventory, LVEF: left ventricular ejection fraction.
